# Higher Thyroid-Stimulating Hormone, Triiodothyronine and Thyroxine Values Are Associated with Better Outcome in Acute Liver Failure

**DOI:** 10.1371/journal.pone.0132189

**Published:** 2015-07-06

**Authors:** Olympia Anastasiou, Svenja Sydor, Jan-Peter Sowa, Paul Manka, Antonios Katsounas, Wing-Kin Syn, Dagmar Führer, Robert K. Gieseler, Lars P. Bechmann, Guido Gerken, Lars C. Moeller, Ali Canbay

**Affiliations:** 1 Department of Gastroenterology and Hepatology, University Hospital, University Duisburg, Essen, 45122, Essen, Germany; 2 Regeneration and Repair Group, The Institute of Hepatology, London, WC1E 6HX, United Kingdom; 3 Liver Unit, Barts Health NHS Trust, London, United Kingdom; 4 Department of Endocrinology and Metabolism, University Hospital, University Duisburg, Essen, 45122, Essen, Germany; 5 Rodos BioTarget GmbH, Medical Park Hannover, 30625, Hannover, Germany; Bambino Gesu' Children Hospital, ITALY

## Abstract

**Introduction:**

Changes in thyroid hormone levels, mostly as non-thyroidal illness syndrome (NTIS), have been described in many diseases. However, the relationship between acute liver failure (ALF) and thyroid hormone levels has not yet been clarified. The present study evaluates potential correlations of select thyroid functional parameters with ALF.

**Methods:**

84 consecutively recruited ALF patients were grouped according to the outcome of ALF (spontaneous recovery: SR; transplantation or death: NSR). TSH, free thyroxine (fT4), free triiodothyronine (fT3), T4, and T3 were determined.

**Results:**

More than 50% of patients with ALF presented with abnormal thyroid parameters. These patients had greater risk for an adverse outcome than euthyroid patients. SR patients had significantly higher TSH, T4, and T3 concentrations than NSR patients. Albumin concentrations were significantly higher in SR than in NSR. *In vitro* T3 treatment was not able to rescue primary human hepatocytes from acetaminophen induced changes in mRNA expression.

**Conclusions:**

In patients with ALF, TSH and total thyroid hormone levels differed significantly between SR patients and NSR patients. This might be related to diminished liver-derived transport proteins, such as albumin, in more severe forms of ALF. Thyroid parameters may serve as additional indicators of ALF severity.

## Introduction

Changes in thyroid-stimulating hormone (TSH) and thyroid hormone levels are often present in patients with severe diseases. Such changes are seen as a consequence of the illness and are therefore called ‘non-thyroidal illness syndrome’ (NTIS) and have been described in many diseases including myocardial infarction, heart failure or sepsis [1–3]. NTIS is not precisely defined and encompasses changes in serum thyroid hormone concentrations following any acute or chronic illness not caused by an intrinsic abnormality in thyroid function. The syndrome is typically associated with low total triiodothyronine (TT3) levels. Increasing severity of the underlying illness correlates with an additional decrease in total thyroxin (TT4) levels. Usually, TSH is also reduced or within the reference range [4]. Up to now it is unclear whether NTIS represents either an adaptive mechanism to reduce energy expenditure and stress or must be considered as pathologic condition [5]. Thyroid hormone replacement therapy has been evaluated in many studies, mostly in patients suffering from cardiac diseases [6]. The presence of low T3 syndrome has been described as an adverse prognostic factor in heart failure after myocardial infarction [1] as well as in intensive care patients with liver cirrhosis [7] in whom reduced free T3 (fT3) and free thyroxine (fT4) levels were found associated with increased mortality. Severe disease states thus affect thyroid function and, conversely, the severity of disease may be affected by pre-existing alterations of thyroid function.

We here focus on the role of thyroid parameters in acute liver failure (ALF) [8]; although a life-threatening critical illness, the association of thyroid hormone levels and outcome in ALF has not yet been examined. Data on thyroid parameters in human ALF is virtually absent. Moreover, conflicting results have been published on thyroid dysfunction in the setting of various liver diseases. On the one hand, hypothyroidism has been associated with increased liver enzymes, and myxedema can complicate or mask hepatic encephalopathy [9, 10]. Hyperthyroidism on the other hand – in its most extreme form as a thyroid storm – can be the primary cause of ALF, either directly or indirectly due to the hepatotoxicity of thyreostatic drugs [11, 12]. In a small retrospective study, Oren *et al*. found a significant negative correlation between TSH blood levels and clinical deterioration in patients with liver cirrhosis that manifested as bleeding varices, development of ascites, and episodes of encephalopathy; these findings suggest that a mild controlled decreased thyroid function might be beneficial for cirrhotic patients [13]. Moreover, Orrego *et al*. suggested propylthiouracil as an antithyroid medication to improve the survival in patients with alcoholic liver disease [14]. In portal vein-ligated rats, hypothyroidism decreased portal venous inflow and portal pressure [15], and hyperthyroidism could aggravate liver fibrosis in a rat model of thioacetamide-induced cirrhosis [16]. Another group showed that T3 can increase the rate of hepatocyte proliferation and lead to a more rapid return to normal liver mass in a 70% hepatectomy rat model [17]. Upon thioacetamide-induced liver failure, the ability of rat hepatocytes to proliferate in response to fT3 was maintained during severe acute toxic injury [18].

In a study of Kostopanagiotou *et al*. involving a pig model of ALF, serum T3 and T4 levels markedly decreased after induction of ALF, whereas fT3 and fT4 levels did not change [19]. In summary the above described data suggests that ALF leads to changes of thyroid serum parameters. In the present study, we thus aimed to analyze ALF patients (i) for thyroid function and (ii) for a potential correlation of thyroid parameters with the course and/or outcome of disease already at initial presentation. (iii) In addition the impact of T3 on acetaminophen induced cell death in primary human hepatocytes was evaluated *in vitro*.

## Material and Methods

### Patient cohort

This retrospective study was carried out in accordance with the Declaration of Helsinki and the guidelines of the International Conference for Harmonization for Good Clinical Practice. It was approved by the local Ethics Committee at the University Hospital Essen (Institutional Review Board).

Patients enrolled fulfilled the criteria for ALF established by the German Acute Liver Failure Study Group. These comprise initial hospital presentation within four weeks of disease onset without apparent pre-existing liver disease at admission and an international normalized ratio ≥1.5 with and without encephalopathy ≥ grade 1 [20]. Patients with liver failure due to cardiac failure and patients with previously known liver disease were excluded. Other causes of liver dysfunction were excluded, such as acute-on-chronic liver failure or pre-existing cirrhosis. Upon admission, clinical data were collected and serum samples were acquired. Outcome was defined by status after four weeks post admission as either spontaneous recovery (SR) or non-spontaneous recovery (NSR) due to either transplantation or death.

### Thyroid function assessment

Thyroid function parameters TSH, fT3, fT4, TT3 and TT4 were determined in serum taken on hospital admission with automated immunoassays (Advia Centaur XP, Siemens Healthcare Diagnostics, Eschborn, Germany, normal ranges provided in Table 1). Patients were grouped according to thyroid function as defined in S1 Table.

### Hepatocyte isolation, culture, and stimulation

Liver tissue samples were retrieved from 3 patients undergoing liver surgery for metastases. Liver tissue was precut from an area as far as possible from any metastases or macroscopic alterations of the liver surface as possible. For isolation of human primary hepatocytes (PHH) resected liver segments were perfused with HBSS (PAA, Pasching, Austria) with EGTA to wash out remaining blood followed by HBSS with 240–250 U/ml collagenase type IV (Sigma-Aldrich, Steinheim, Germany) to digest connective tissue. The obtained cell suspension was filtered through a 4μm mesh and washed 3 times in HBSS. The cell pellet was resuspended in cell culture medium (DMEM/Ham’s F-12 with 10% heat-inactivated FCS stripped of Thyroid hormones by anion exchange resin as previously described [21], 100U/ml penicillin, 0.1mg/ml streptomycin and 2mM L-glutamine; PAA, Pasching, Austria) and seeded at a density of approx. 1 x 10^6^ cells/cm^2^. Cells were kept in Thyroid cell culture medium containing hormone-deprived FCS for 48h prior to T3 stimulation. To mimic an ALF-like condition acetaminophen (Sigma-Aldrich; Steinheim, Germany) was added to a final concentration of 5mM to the cells for 24h with or without T3 in a final concentration of 2x10^-9^ M. This corresponds to 3–5 times of the physiological levels as described previously by Moeller et al [21], suggesting clinically relevant effects by stimulation with this concentration. Control cells were treated with vehicle (Ethanol).

To induce apoptotic cell death cells were incubated with the CD95/Fas-agonist CH11 (anti-Fas (human, activating) clone CH11; Millipore, Temecula, CA, USA) in a final concentration of 100ng/ml for 5 hours again with or without T3. Hepatocytes were washed twice with ice-cold PBS and lysed for 15 min at 4°C in mRIPA buffer (50 mM Tris-HCL, pH 7.5, 150 mM NaCl, plus protease inhibitors). Protein lysates were centrifuged at 13,000g for 15 min at 4°C and total protein concentration in the supernatant was determined using BCA assay (Thermo Scientific; Rockford, IL, USA). The apoptosis marker M30, which recognizes caspase-cleaved cytokeratin 18 filaments derived from apoptotic cells [22–26], was assessed in equal amounts of whole protein lysates (20μg) using the M30-Cytodeath ELISA Kit (Peviva; Nacka, Sweden).

### RNA isolation and qrt PCR

At the end of the stimulation period total RNA extraction and purification was performed using RNeasy Mini Kit (Qiagen; Hilden, Germany) following manufacturer instructions. Reverse transcription was performed with the QuantiTect RT kit (Qiagen; Hilden, Germany) using 1μg of total RNA.

Qrt-PCR for specific mRNA sequences was performed on a CFX96 Touch Real-Time PCR Detection System (Biorad, Munich, Germany) using QuantiTect SYBR Green Kit (Qiagen; Hilden, Germany) in a volume of 15μl including 2μl of cDNA. Oligonucleotide sequences used as primers are given in S2 Table. Melting curves were collected to ascertain specificity of PCR products. Changes in mRNA expression were calculated by the ΔΔ-ct method and are presented as foldchanges in relation to expression of a reference gene (hypoxanthine-guanine phosphoribosyltransferase, HPRT) in vehicle-treated cells.

### Statistical analysis

SPSS 17.0 for Windows (SPSS Inc., Chicago, IL, USA) was used for statistical analysis. Normality was assessed with Shapiro-Wilk test. For comparison between groups, two-tailed *t*-tests or nonparametric tests (Mann-Whitney U) were used. Univariate logistic regression analysis was used to identify predictive value of a parameter. To adjust for several risk factors, multivariate logistic analysis was performed with all the variables found significant in univariate analysis in a single step. Statistical significance for *in vitro* experiments was determined by one-way ANOVA (with Tukey’s post-hoc test for individual experimental conditions), performed with Prism 5 (GraphPad Software, Inc.; La Jolla, CA, USA). Data are presented as mean values ± SEM. Differences were considered significant at *p*<0.05.

## Results

### Patient cohort

In total, 84 patients (53.6% females / 46.4% males; ages 40.4 ± 1.7 years) with ALF were enrolled in this retrospective study. The main underlying etiologies for ALF were drug toxicity, hepatitis B and autoimmune hepatitis (detailed distribution of the etiologies is shown in S1 Fig). Clinical parameters at admission are presented in Table 1. Sixty-one patients (72.5%) recovered under conservative therapy and were grouped as SR. Eleven patients (15%) died without transplantation, and twelve patients (12.5%) received a liver transplant. Two patients died after liver transplantation. Deceased and transplanted patients were grouped as NSR.

**Table 1:** ALF patients: Clinical parameters.

Parameter	Mean ±SEM	Reference range
TSH (mIU/L)	1.1±0.1	0.3–3
fT4 (pmol/L)	15.7±0.5	11.5–22.7
fT3 (pmol/L)	3.4±0.1	3.5–6.5
TT4 (nmol/L)	147.5±8.2	58–161
TT3 (nmol/L)	1.4±0.1	0.9–2.8
TPZ%	42.97±1.82	70–130
INR	2.0±0.1	-
aPTT (sec)	37.9±1.6	27–37
Fibrinogen (mg/dl)	242.43±10.0	180–350
Creatinine (mg/dl)	1.4±0.1	0.9–1.3
Calcium (mmol/l)	2.1±0.0	2.15–2.5
Phosphate (mg/dl)	3.2±0.1	2.7–4.5
Bilirubin (mg/dl)	13.3±1.2	0.3–1.2
AST (U/l)	3141.9±389.3	<50
ALT (U/l)	3026.0±285.0	<50
AP (U/l)	161.0±10.8	25–124
GGT (U/l)	179.5±17.5	<55
GLDH (U/l)	608.9±147.7	<7
Ammonia (±mol/L)	137.2±21.2	19–55
Albumin (g/dL)	3.3±0.1	3.4–4.8

Six patients were on thyroxine substitution therapy; one of these was euthyroid, three had otherwise abnormal thyroid parameters and two suffered from iatrogenic hyperthyroidism. Low TT3 was detected in 28 patients (33.3%), 9 of these had low TT3 and TT4 (10.7%). Twenty two patients (26.2%) had normal TT3 and TT4, 29 patients had normal TT3 and high TT4 (34.5%), one patient had high TT3 and normal TT4 (1.2%) and 4 patients had both high TT3 and TT4 (4.8%). The distribution of outcome according to thyroid status is shown in Table 2. The incidence of hyperthyroidism was significantly higher in the NSR than in the SR group. Moreover the incidence of abnormal thyroid hormone levels was higher in ALF patients than in the general population (Table 3). To assess if the signaling axis between pituitary gland and thyroid was affected by ALF, growth hormone levels in serum were assessed. Though, no differences between SR and NSR were found (S2 Fig).

**Table 2:** Grouped outcome and thyroid status.

Thyroid status	NSR^1^ *	SR^2^ *
Euthyroidism^#^	4 (14%)	24 (86%)
Other abnormal thyroid hormone values^#^	13 (28%)	33 (72%)
Hyperthyroidism^#^	5 (83%)	1 (16%)
Hypothyroidism^#^	1 (50%)	1 (50%)

^1^NSR = non-spontaneous remission.

^2^SR = spontaneous remission.

* Distribution of thyroid status differed significantly between the groups (*p* = 0.005).

^#^ Percentages are given for the total numbers of patients in each group of thyroid function.

**Table 3:** Thyroid function and parameters in our patient collective and in the general population in Germany (28).

	Presented collective	General Population	
Hypothyroidism	1.2%	0.9%	
Hyperthyroidism	7.3%	0.6%	
		General Population (men)	General Population (women)
TSH (mIU/L)	1.06 ± 0.11	1	0.9
TT4 (nmol/L)	147.5 ± 8.2	87.52	93.95
TT3 (nmol/L)	1.44 ± 0.09	2	2

### Are individual serum parameters of thyroid function associated with ALF outcome?

**Fig 1:**
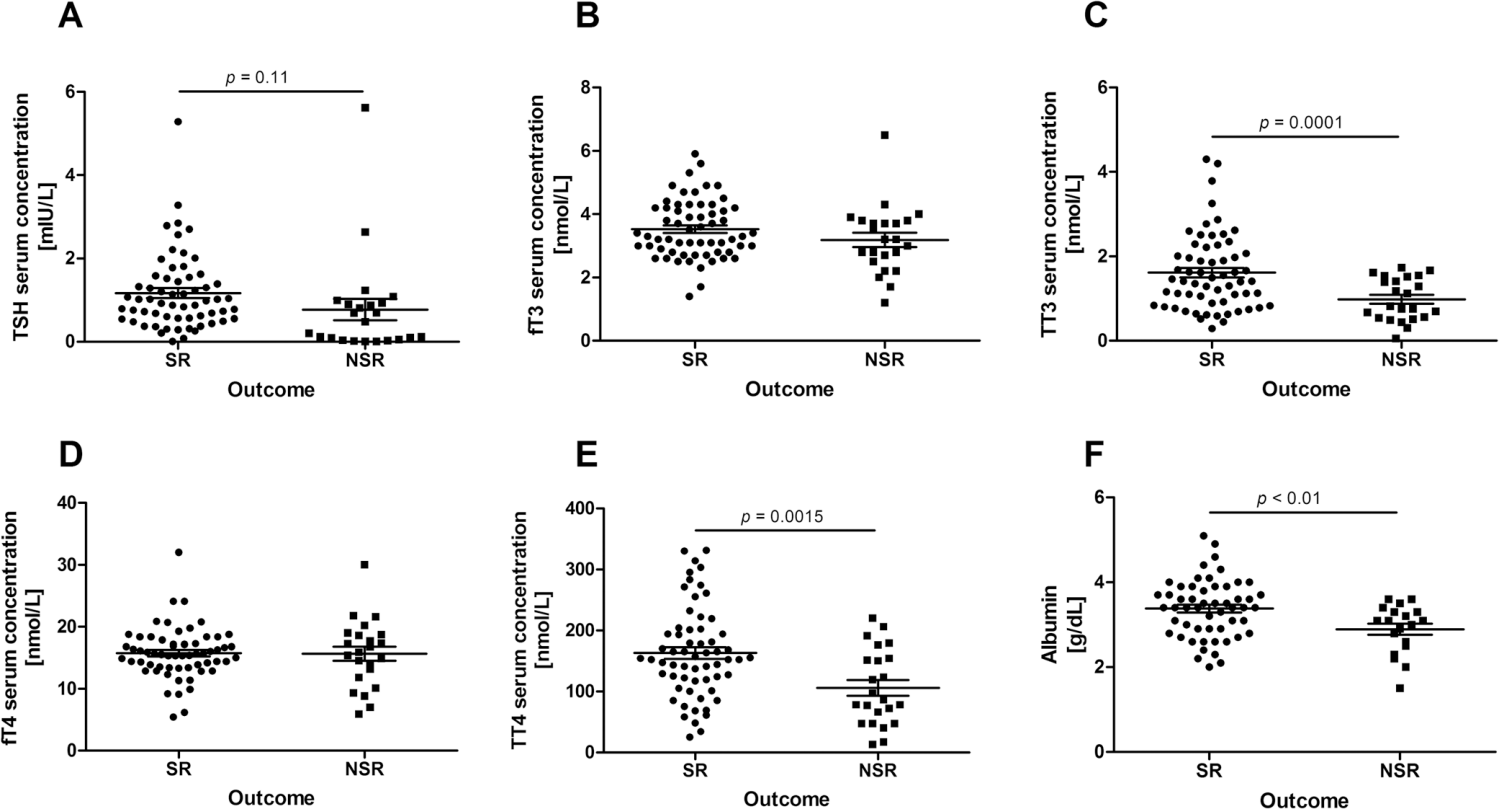
Distribution of thyroid function parameters in ALF. Individual parameters are plotted for the spontaneous remission (SR) and for the non-spontaneous remission (death or liver transplantation) patients with ALF. TSH (A), total T3 (TT3, C), and total T4 (TT4, E) were significantly lower in the NSR group. Free T3 (fT3, B) and free T4 (fT4, D) did not differ between the groups.

**Fig 2:**
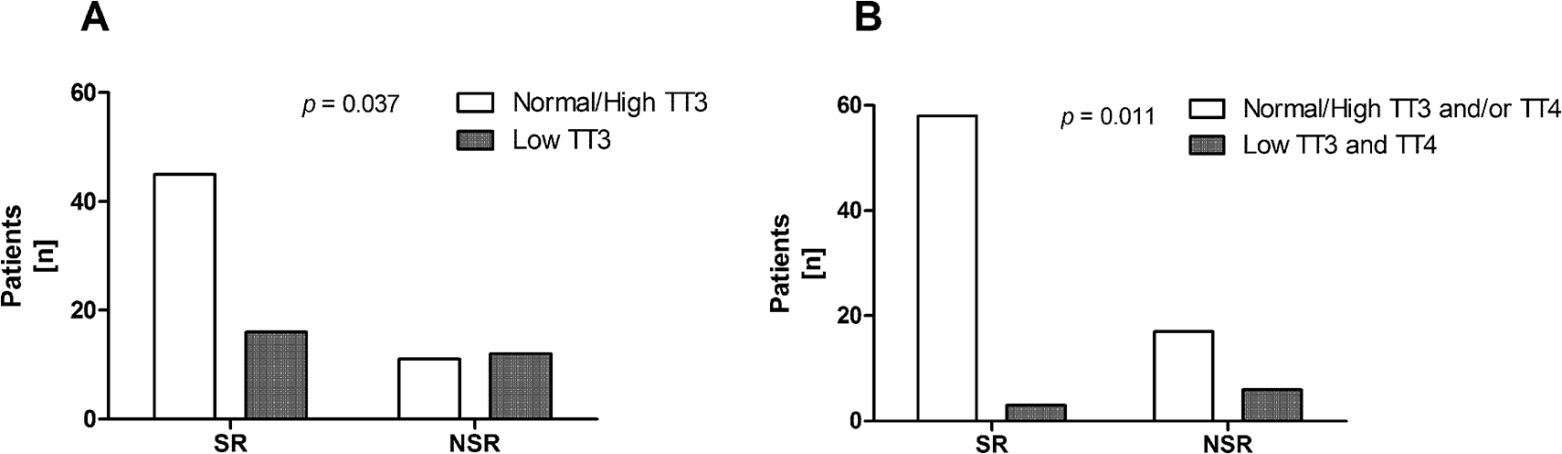
Outcome according to TT3 status. Incidence of low serum triiodothyronine for the spontaneous remission (SR) and for the non-spontaneous remission (death or liver transplantation) patients with ALF is shown. Low serum total T3 (TT3, A) alone occurred significantly more often in the NSR group compared to SR. Similarly low TT3 in combination with low total T4 (TT4, B) was present in a higher proportion of NSR patients than in SR.

Individual serum concentrations of thyroid parameters were analyzed for differences between SR and NSR. TSH, TT4, and TT3 were significantly higher in the SR group (Fig 1A, 1C and 1E), while there was no significant difference in the free hormone concentrations fT4 and fT3 (Fig 1B and 1D). In addition, albumin was significantly lower in the NSR group than in SR patients (Fig 1F). Incidence of low albumin (< 3.4g/dl) was significantly higher in NSR patients (78.9%) than in the SR group (40.7%; *p* = 0.005). Prevalence of low TT3 or low TT3 and TT4 was significantly higher in the NSR group (Fig 2A and 2B). The degree of total thyroid hormone reduction appears to correlate with the prognosis of ALF.

### Prognostic relevance of thyroid parameters for ALF outcome

**Fig 3:**
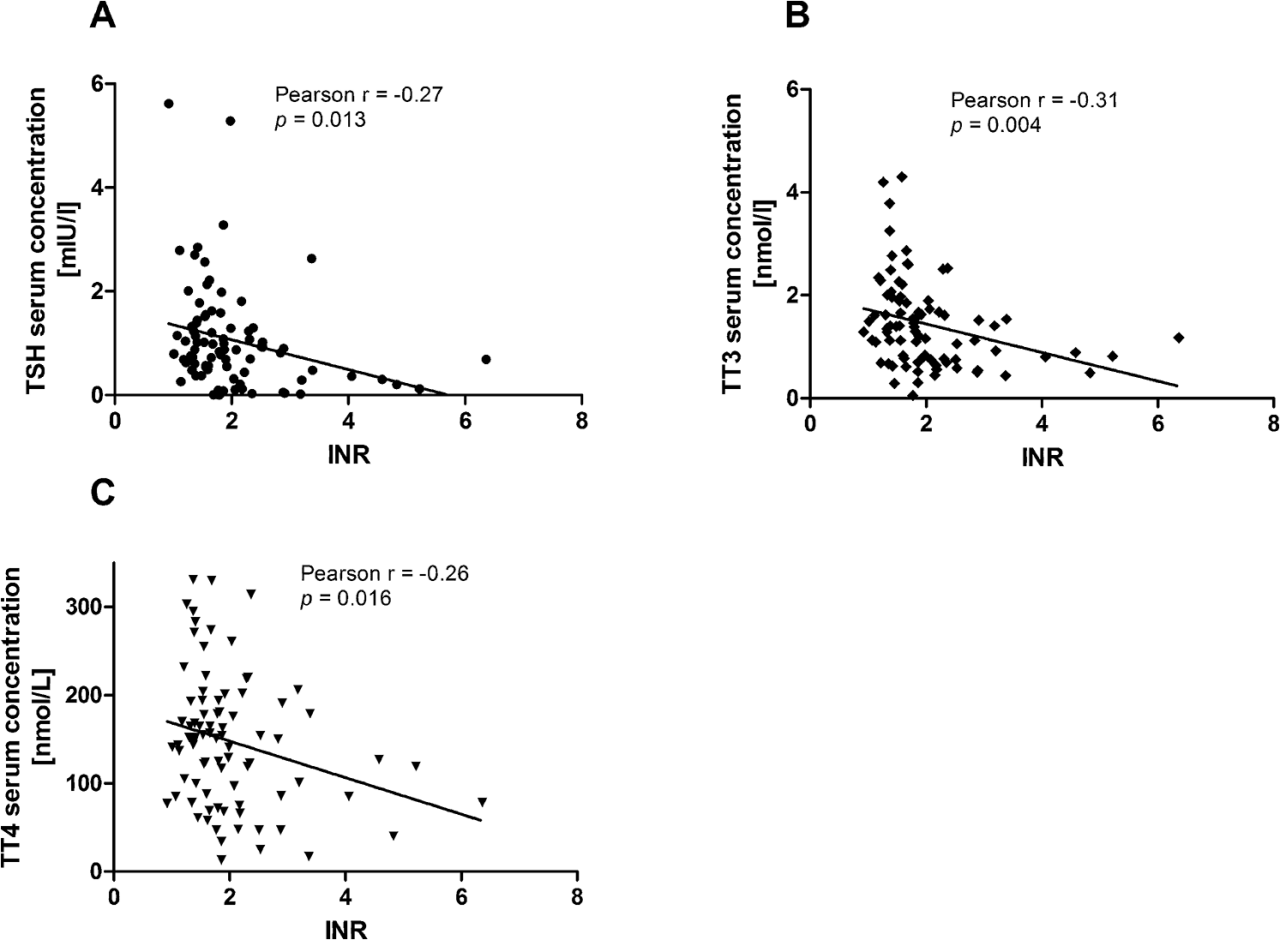
Correlation of serum thyroid parameters to the international normalized ratio (INR). INR served as surrogate marker for liver function. TSH (A), TT3 (B), and TT4 (C) were weakly, inversely correlated to INR.

Univariate regression analyses were performed with outcome as dependent variable with all thyroid parameters. This resulted in a significant correlation between recovery and TSH (regression coefficient -0.789, *p* = 0.016, OR 0.454), recovery and TT4 (regression coefficient -0.013, *p* = 0.003, OR 0.987) and recovery and TT3 (regression coefficient -1.318, *p* = 0.004, OR 0.268). There was no significant correlation between recovery and fT4 or fT3. Multivariate logistic analysis, with the final model including TSH and TT4, showed a significant correlation between the parameters mentioned above and recovery. For TSH, the regression coefficient reached -0.652 (*p* = 0.066) and the odds ratio for recovery reached 0.521. The regression coefficient for TT4 was -0.014 (*p =* 0.002), with an odds ratio for recovering of 0.986. In addition TSH, TT3, and TT4 were inversely correlated to international normalized ratio (INR) as surrogate measure of liver function (Fig 3A–3C). TT3 and TT4 were inversely correlated to the prevalence of hepatic encephalopathy (TT3: Spearman r = -0.3993, p < 0.001; TT4: r = -0.3984, p < 0.001).

### T3 cannot rescue primary human hepatocytes from acetaminophen induced damage

**Fig 4:**
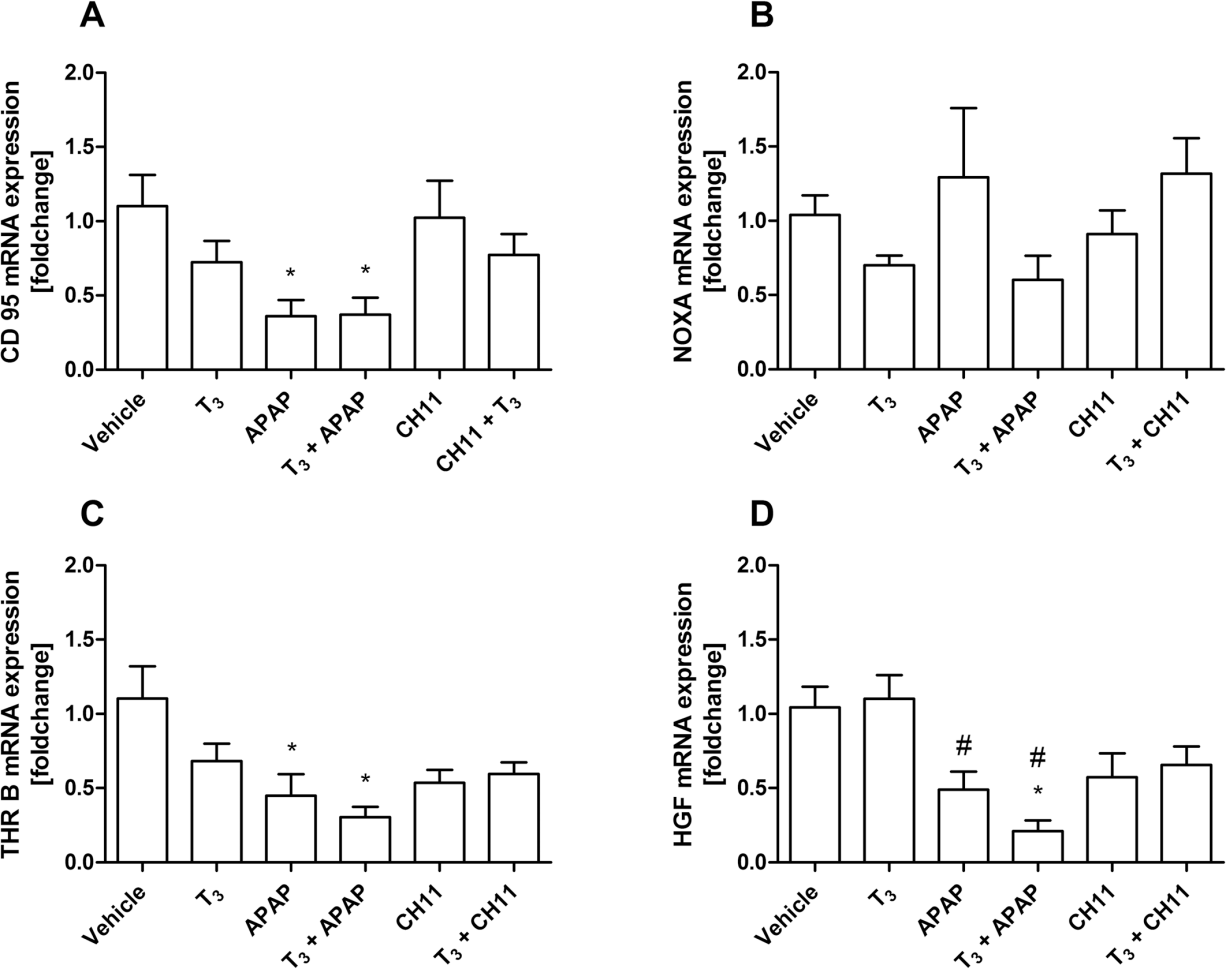
Gene expression in primary human hepatocytes after toxic injury and T3 stimulation. mRNA expressions of cell death related genes CD95 (A) and NOXA (B), as well as the thyroid hormone receptor β1 (THR B, C) and hepatocyte growth facor (HGF, D) were assessed. APAP significantly reduced mRNA expressions of CD95 (Fas receptor), THR B, and HGF. * *p* < 0.05 compared to vehicle (ethanol); # *p* < 0.05 compared to T3. Statistical significance experiments was determined by one-way ANOVA with Tukey’s post-hoc test for individual experimental conditions. APAP: acetaminophen; CH11: CD95/FAS-agonist to induce apoptosis; T3: triiodothyronine.

To investigate if T3 may be able to diminish cellular injury PHH isolated from 3 different donors were treated with acetaminophen (APAP) or CH11 to induce necrotic or apoptotic cell death, respectively. T3 treatment was performed in parallel to the cell injury, to mimic the condition of ALF, where cell death is already established upon diagnosis. Activation of thyroid hormone receptor signaling by T3 was checked by mRNA expression of deiodinase 1 (DIO1). T3 stimulation resulted in significant upregulation of DIO1 mRNA (see S3 Fig). APAP or CH11 treatment did not lead to macroscopically observable cell death in PHH. Though, CH11 treatment led to an increase of M30 (a caspase-cleaved cytokeratin18 fragment; data not shown). Expression of the Fas receptor CD95 was significantly reduced in APAP treated cells compared to vehicle, irrespective of T3 stimulation (Fig 4A). NOXA mRNA, serving as an early marker of apoptosis, was not affected by any treatment condition (Fig 4B). Similar to CD95 mRNA, expression of the thyroid hormone receptor β1 (THR B, Fig 4C) and of hepatocyte growth factor (HGF, Fig 4D) were significantly diminished by APAP compared to vehicle. In summary T3 stimulation did not affect APAP induced reduction of cell death related or growth factor mRNA expression.

## Discussion

Up to now, detailed data on thyroid parameters in the setting of acute liver failure was not available. The present study expands current knowledge by demonstrating that more than half of the enrolled patients with ALF display abnormal thyroid parameters indicating an interaction of thyroid and liver during this condition. The prevalences of hypo-, hyperthyroidism and other abnormalities in the ALF cohort differ from those observed in the general population [27]. The main result was that patients who recovered from ALF had higher TSH, TT4, and TT3 levels than patients who required liver transplantation or died from ALF. TSH and TT4 were both found positively associated with recovery from ALF. Moreover, prevalence of low TT3 or combined low TT3/TT4 was significantly higher in the NSR group than among SR patients.

While thyroid status has been previously investigated in critically ill patients or chronic liver diseases, the above described results represent the first systematic evaluation in ALF. Similar findings were described for intensive care unit patients of various causes including fulminant hepatic failure [28]. TT3 and TT4 concentrations, but not fT3 and fT4, were significantly lower in survivors than in non-survivors in this critically ill collective. Low TSH has also been associated with critical illness [29] whereas elevated TSH indicated recovery [30]. A similar trend was observed in our ALF collective, though without reaching significance. The most common finding in NTIS is decreased serum TT3, in more severe forms also TT4 and TSH are diminished. NTIS in general seems to be associated with poor prognosis [5]. According to this definition NTIS was present in a substantial proportion of the presented ALF patients at hospital admission and was associated with adverse outcome. Since growth hormone levels did not differ between the groups and exhibited a pattern suggesting pulsative release, as in healthy individuals, we do not expect a hormonal dysregulation upstream of the thyroid.

Various studies in chronic liver diseases have been performed on thyroid serum parameters. In alcoholic liver disease TSH increased with progressing liver dysfunction [31] and T3 levels were inversely correlated with the severity of alcoholic liver disease [31, 32]. Reduced fT3 and fT4 levels were associated with increased mortality in patients with liver cirrhosis [7], and clinical deterioration of liver cirrhosis was negatively correlated to TSH blood levels [13]. Hepner *et al*. reported that serum T3 and T4 levels were lower in cirrhotic patients who died within three months compared to survivors [33]. In contrast, patients who survived liver transplantation had higher T4 and lower T3 levels during the evaluation process and a much smaller T3/T4 ratio than those who died during evaluation or after transplantation [34]. Though, in our patient collective, T3/T4 ratio did not differ between SR and NSR patients (data not shown). One study compared patients with fulminant hepatitis, patients with chronic liver disease and healthy control subjects, where the lowest serum T3 values were found in fulminant hepatitis [35]. Collectively these studies and our own results indicate low T3 concentration to be associated with adverse disease courses in chronic and acute liver diseases.

Although total hormone levels were changed significantly in the presented ALF cohort, free thyroid hormones did not differ between SR and NSR patients. This finding is in line with ALF in a pig model, where serum thyroxin T3 and T4 levels markedly decrease after ALF induction, whereas fT3 and fT4 levels did not change [19]. Reduction of total but not free hormone concentrations in NSR compared to SR patients might imply a deficit of transport proteins produced by the liver (i.e. thyroid hormone-binding globulin (TBG) or albumin), which are crucial for thyroid hormone function [36]. This has been described in patients receiving bypass surgery, where TBG levels diminished rapidly after surgery [37]. In contrast, increased TBG levels in chronic liver disease have been reported previously [38, 39] suggesting that this may not be the main factor for decreased total thyroid hormones in our cohort. However, significantly lower albumin amounts were found in sera of NSR patients compared to SR patients. This could be a sign of a more severe ALF course and may explain reduced TT3 and TT4 concentrations. Thus, severely reduced liver function might impact the concentrations of TT3 and TT4 *via* diminished albumin production. This hypothesis for a possibly involved mechanism for ALF affecting thyroid hormones should be tested in larger cohorts or models of ALF.

Another result of our study is an unfavorable outcome in patients with hyperthyroidism. Besides the fact that hyperthyroidism increases oxidative stress in rat hepatocytes [40], hyperthyroidism also increases cardiac output without effecting an increase in hepatic blood flow. The combination of increased oxygen consumption and decreased organ perfusion leads to tissue hypoxia, which is assumed to contribute significantly to liver dysfunction [41]. The unfavorable outcome in patients with hyperthyroidism would recommend close monitoring of patients presenting with ALF.

Treatment with thyroid hormones in liver disease has been evaluated in numerous studies (for overview, see Chi *et al*. [42]). However, the findings are partially conflicting and difficult to interpret due to the variety of settings, patient collectives, or animal models, respectively. T3 has been shown to further hepatocyte proliferation after partial hepatectomy or after toxin-induced liver failure [17, 18]. Fernandez *et al*. reported that T3 administration exerted significant protection against ischemia-reperfusion injury in the rat [40]. In contrast, hyperthyroidism enhanced Kupffer cell function contributing to increased oxidative stress in a rat model [43]. Thyroxin administration reduced the pre-ischemic and postreperfusion concentrations of adenosine triphosphate and glutathione in the liver, thus increasing the susceptibility of isolated rat hepatocytes to anoxia and oxidative stress [44]. In the presented manuscript, we aimed to mimic the condition of acute liver failure by treating primary human hepatocytes with T3 in parallel to toxic or apoptotic injury. In this setting T3 was not able to restore reduced gene expression affected by APAP. It could be possible that T3 exerts a protective effect, reducing damage to hepatocytes but may have no effect on ongoing or established injury. Although low serum T3 may indicate more severe forms of liver diseases or even could aggravate liver injury, application of T3 in liver disease settings seems not generally warranted, as the underlying mechanisms of the liver-thyroid axis are not well understood, yet.

In summary, we present the first systematic evaluation of the thyroid hormone status in patients with ALF to our knowledge. More than 50% of ALF patients in this study exhibited abnormal thyroid parameters, which had worse outcomes compared to euthyroid patients. Patients who recovered without liver transplantation had higher TSH, TT4 and TT3 concentrations, while there was no difference in free hormone concentrations between the groups. In conclusion, the total thyroid hormone levels, in particular TT3, may be of prognostic value for survival without liver transplantation in ALF. A possible mechanism linking liver function and thyroid hormones may be reduced availability of albumin as binding protein for thyroid hormones. This important finding warrants further studies to investigate the mechanisms underlying this effect.

## Supporting Information

S1 Fig Etiology distribution of the ALF patient cohort. (TIF)

S2 Fig Growth hormone serum concentrations. To investigate if the altered thyroid hormone levels may be due to disturbed signaling from the pituitary gland, growth hormone (GH) concentrations were detected. Clustering of the concentrations at high and low amounts, suggest an undisturbed pulsative release of GH by the pituitary. Furthermore, no differences between the groups were detected.
(TIF)

S3 Fig Activation of thyroid signaling pathways in primary human hepatocytes. Primary human hepatocytes were stimulated with T3 for 24h. mRNA expression of deiodinase 1 (DIO1), a gene transcribed upon activation of thyroid hormone receptors, was measured to ascertain stimulation of hepatocytes by T3. DIO1 mRNA expression was significantly increased in cells with T3 treatment compared to vehicle (ethanol).
(JPG)

S1 Table Definitions of thyroid functional status. (DOC)

S2 Table Oligonucleotides used as primers for qrtPCR. (DOCX)
