# Supplementary figures and images for: Higher Thyroid-Stimulating Hormone, Triiodothyronine and Thyroxine Values Are Associated with Better Outcome in Acute Liver Failure
Source: PLoS One. 2015 Jul 6;10(7):e0132189. doi: 10.1371/journal.pone.0132189 (PMC4493082; doi:10.1371/journal.pone.0132189)

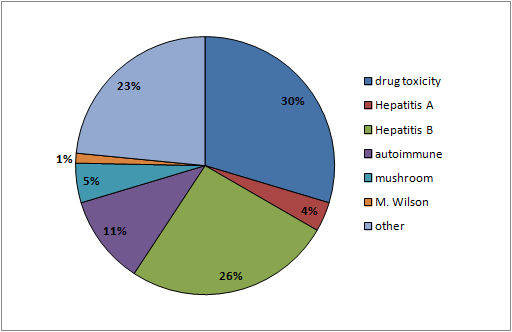

Supplement: S1 Fig — (TIF) [file pone.0132189.s001.tif]

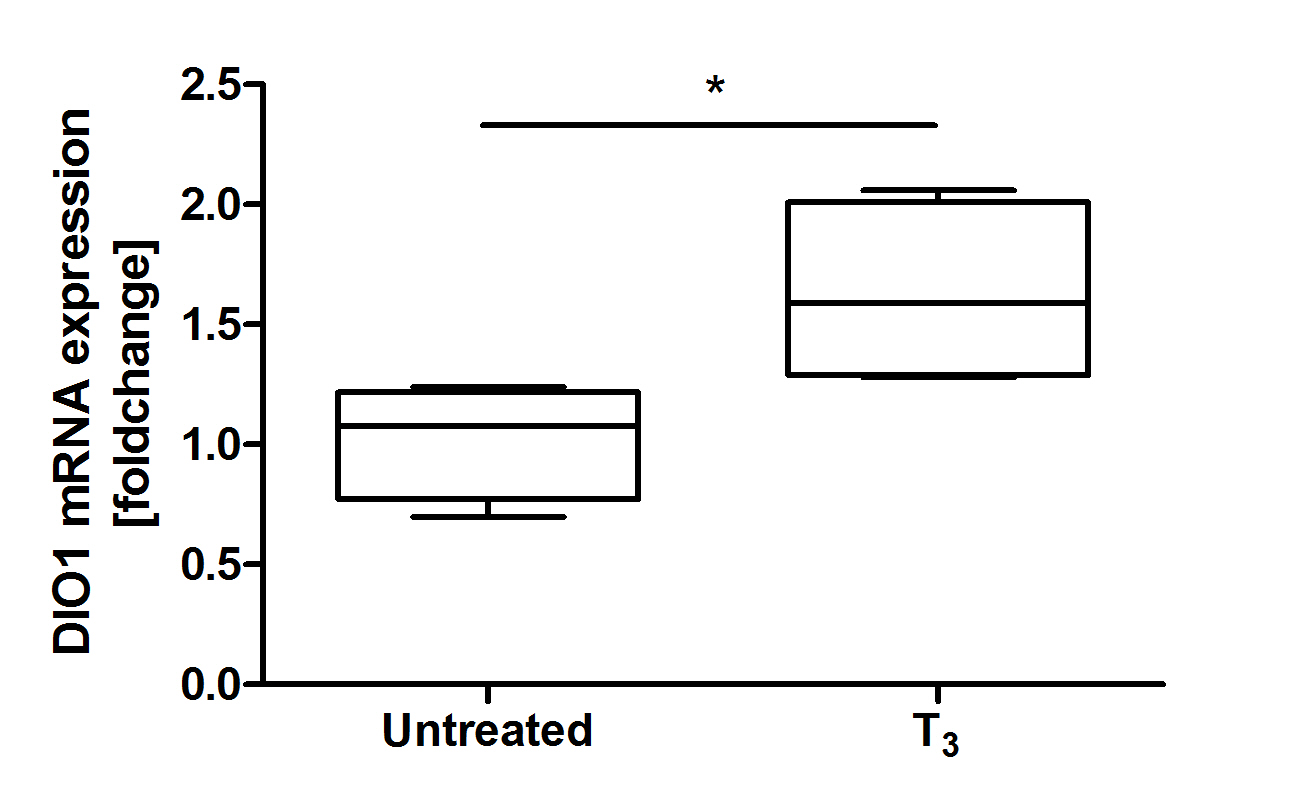

Supplement: S3 Fig — Primary human hepatocytes were stimulated with T3 for 24h. mRNA expression of deiodinase 1 (DIO1), a gene transcribed upon activation of thyroid hormone receptors, was measured to ascertain stimulation of hepatocytes by T3. DIO1 mRNA expression was significantly increased in cells with T3 treatment compared to vehicle (ethanol). (JPG) [file pone.0132189.s003.jpg]
