# Supplementary material for: Higher Thyroid-Stimulating Hormone, Triiodothyronine and Thyroxine Values Are Associated with Better Outcome in Acute Liver Failure
Source: PLoS One. 2015 Jul 6;10(7):e0132189. doi: 10.1371/journal.pone.0132189 (PMC4493082; doi:10.1371/journal.pone.0132189)
Supplement: S1 Table — (DOC) [file pone.0132189.s004.doc]

**Higher thyroid-stimulating hormone, triiodothyronine and thyroxine values are associated with better outcome in acute liver failure**

**Supplemental Table 1. Definitions of thyroid functional status.**

| **Thyroid status** | **TSH** | **fT4** | **fT3** |
| --- | --- | --- | --- |
| Euthyroidism | 0.3-3 mlU/L | 11.5-22.7 pmol/L | 3.5-6.5 pmol/L |
| Hypothyroidsm | > 3 mlU/L | ≤ 22.7 pmol/L | < 3.5 pmol/L |
| Subclinical hypothyroidism | > 3 mlU/L | 11.5-22.7 pmol/L | 3.5-6.5 pmol/L |
| Hyperthyroidism | < 0.3 mlU/L | > 22.7 pmol/L | ≥ 3.5 pmol/L |
| Subclinical hyperthyroidism | < 0.3 mlU/L | 11.5-22.7 pmol/L | 3.5-6.5 pmol/L |
