# Supplementary material for: Higher Thyroid-Stimulating Hormone, Triiodothyronine and Thyroxine Values Are Associated with Better Outcome in Acute Liver Failure
Source: PLoS One. 2015 Jul 6;10(7):e0132189. doi: 10.1371/journal.pone.0132189 (PMC4493082; doi:10.1371/journal.pone.0132189)
Supplement: S2 Table — (DOCX) [file pone.0132189.s005.docx]

**Higher thyroid-stimulating hormone, triiodothyronine and thyroxine values are associated with better outcome in acute liver failure**

**Supplemental table 2:** Oligonucleotides used as primers for qrtPCR.

| **Gene name** | **Oligonucleotide sequence** | **Annealing temperature** |
| --- | --- | --- |
| Hypoxanthine-guanine transferase (HPRT; reference gene) | Forward: 5´-GAC CAG TCA ACA GGG GAC AT-3´  Reverse: 5´-CTT GCG ACC TTG ACC ATC TT-3´ | 52°C |
| CD95/FAS | Forward: 5´-CAA AGC CCA TTT TTC TTC CA-3´  Reverse: 5´-TTT GGT TTA CAT CTG CAC TTG G-3´ | 52°C |
| Deiodinase 1 (DIO1) | Forward: 5´-GCC ACT GGT GCT GAA TTT TGG-3´  Reverse: 5´-GGC CAG ATT TAC CCT GAT GC-3´ | 52°C |
| Hepatocyte growth factor (HGF) | Forward: 5'-ACC ACA CGA ACA CAG CTT TTT GCC-3'  Reverse: 5'-GGG TCC CCC TTC TTC CCC TCG-3' | 52°C |
| NOXA | Forward: 5´-GGA GAT GCC TGG GAA GAA GG-3´  Reverse: 5´-ACT CGA CTT CCA GCT CTG C-3´ | 52°C |
| Thyroid hormone receptor β1 | Forward: 5´-CAG GCA AGG CAG CTA CCA A-3´  Reverse: 5´-TTC ACT GAC ATC TCC TTA CTT CCA-3´ | 52°C |
